# Mitochondria in the Aging Muscles of Flies and Mice: New Perspectives for Old Characters

**DOI:** 10.1155/2016/9057593

**Published:** 2016-08-18

**Authors:** Andrea del Campo, Enrique Jaimovich, Maria Florencia Tevy

**Affiliations:** ^1^Centro de Estudios Moleculares de la Célula, ICBM, Facultad de Medicina, Universidad de Chile, 8389100 Santiago, Chile; ^2^Centro de Genómica y Bioinformática, Facultad de Ciencias, Universidad Mayor, 8580000 Santiago, Chile

## Abstract

Sarcopenia is the loss of muscle mass accompanied by a decrease in muscle strength and resistance and is the main cause of disability among the elderly. Muscle loss begins long before there is any clear physical impact in the senior adult. Despite all this, the molecular mechanisms underlying muscle aging are far from being understood. Recent studies have identified that not only mitochondrial metabolic dysfunction but also mitochondrial dynamics and mitochondrial calcium uptake could be involved in the degeneration of skeletal muscle mass. Mitochondrial homeostasis influences muscle quality which, in turn, could play a triggering role in signaling of systemic aging. Thus, it has become apparent that mitochondrial status in muscle cells could be a driver of whole body physiology and organismal aging. In the present review, we discuss the existing evidence for the mitochondria related mechanisms underlying the appearance of muscle aging and sarcopenia in flies and mice.

## 1. Introduction

Loss of muscle mass and muscle wasting are clinical symptoms associated with many chronic diseases as well as with the aging process. The loss of muscle mass accompanied by a decrease in muscle strength and resistance which occurs in the elderly is termed sarcopenia. In the population over 65 years of age, this decay in muscle function is particularly associated with increased dependence, frailty, and mortality. In fact, sarcopenia is the main cause of disability among the elderly [1, 2]. As the world population increases life expectancy, the demographic group over 65 years of age is expected to grow substantially worldwide. It is a challenge for the governments and the healthcare systems to promote independence and decrease frailty in the elderly. Several lines of evidence suggest that muscle loss and malfunctioning begin long before there is any clear physical impact in the senior adult [3, 4]. Thus, in order to generate preventive therapies for muscle aging, treatments should be directed to younger age groups. Hence, the need to elucidate the origin and mechanisms which drive muscle aging has become a pressing matter.

The molecular mechanisms underlying muscle aging are of multifactorial etiology [5–7]. Among the mechanisms that contribute to sarcopenia have been described the decrease in physical activity, the decrease in anabolic hormones, and an increase in proinflammatory cytokines as well as the increase in catabolic factors [3, 4]. Further, recent studies have also identified that not only mitochondrial metabolic dysfunction but mitochondrial dynamics and mitochondrial calcium uptake too could be involved in the degeneration of skeletal muscle mass [8, 9]. A growing body of evidence suggests that muscle quality plays a systemic role in the aging process [10–13]. Thus, it has become apparent that mitochondrial status in muscle cells could be a driver of whole body physiology and organism aging.

To better understand and untangle the complexity of the molecular mechanisms driving sarcopenia and the contribution of muscle decay to the integral aging process, more studies using model organisms are required in the future. In the present review, we discuss the existing evidence for the mitochondria related mechanisms underlying the appearance of muscle aging and sarcopenia in flies and mice.

## 2. Mitochondrial Dysfunction and Oxidative Stress in Aged Muscle

Reactive oxygen species (ROS) are produced in the mitochondria as a byproduct of an inefficient transfer of electrons through the Electron Transport Chain (ETC) [14]. During the aging process, ROS production increases as well as mitochondrial damage and dysfunction (Table 1). These phenomena have also been observed in age-associated diseases. In fact, it is supposed that the observed increase in ROS is derived from a decline in mitochondrial function [15]. Interestingly, in flies, the development of genetic sensors which can be targeted specifically to a tissue or to an organelle within the cell is helping to reveal which tissues are subject to redox dysregulation during aging [16]. Increased production of ROS in aged and age-related phenotypes has also been observed to be accompanied by alterations in mitochondrial DNA (mtDNA) quality and quantity [17–20]. It has been proposed that increases in ROS could easily target the mtDNA which lacks histone protection. Furthermore, it is argued that with aging, DNA repair mechanisms efficiency decline and could lead to mutations in mtDNA [21]. In* Drosophila*, it has been observed that naturally occurring variations in mtDNA can have a significant influence on mitochondrial bioenergetics and longevity. Variations in mitochondrial respiration on permeabilized muscle fibers were directly linked to naturally occurring mtDNA haplotypes, even in young flies. As flies aged, an increase in ROS production and copy number of mtDNA were observed in all strains and the percentage increase in each strain could be associated with the mtDNA haplotype and with longevity [22]. These experiments comply with the ruling hypothesis. However, elegant experiments in* Drosophila,* using naturally occurring mtDNA haplotypes in an isogenic nuclear background, have recently shown that mtDNA affects both copy number of mitochondrial genomes and patterns of expression of mitochondrial protein coding genes. Strikingly, these experiments showed that a high expression of ND5 (the mitochondrial complex I NADH-Ubiquinone oxidoreductase) inversely correlates with longevity in males but not in females [23]. Considering that such experiments were performed in whole organisms and no functional experiments were performed on mitochondrial proteins, it will be interesting to study data from purified muscle mitochondria of these flies. Consistent with the paradigm, in mice, it has been found that ROS production is increased in aged muscles and directly affects the complex V (ATP synthase) of the ETC, oxidizing, thereby preventing the synthesis of ATP by the oxidized protein [21]. An increase of 8-oxodeoxiguanosina (8-oxoG) has also been observed, which is one of the markers indicating mtDNA oxidation [24]. One possible consequence of this process is that the damaged mtDNA promotes the biogenesis of damaged mitochondria, in turn producing more ROS, enabling a vicious cycle to continue [25]. Contrasting these results, recent deep sequencing of the C57BI/6 mice mitochondrial genome suggests, otherwise, that mutations in the mtDNA arise from replication errors during early life [26]. New techniques based on Next Generation Sequencing (NGS) [26–28] or multiplex real time PCR [29–31] along with the improvement of bioinformatics tools for analysis of big data have recently been developed in order to attain more accurate measures of the level of deletions and copy number of mtDNA in skeletal muscle. NGS based methods as ultradeep sequencing have a high intrinsic error incurred during library preparation and reading; it is difficult to filter genomic DNA contamination reads from the sample. To circumvent this issue, barcoded libraries and barcoded libraries followed by rolling circle amplification methods are being developed [27, 28]. Such NGS methods coupled to single muscle fiber mtDNA extraction will shed light on to the role of deletions and copy number of mtDNA in tissue specific aging. Other new methods have tried simultaneous amplification of MT-ND4, MT-ND1, and the noncoding D-Loop region by real time PCR in single muscle fibers to detect and quantify mtDNA mutations [31]. These new and more sensitive techniques have yet to be tried in sarcopenic muscle. Correlation of these measurements with ROS levels across lifespan will shed light onto the role of ROS as a mutagenic agent of mtDNA.

Increased ROS species in the cell have also been associated with diminished ROS scavengers activities during aging [32–34] (Table 1). Seminal experiments showed that genetic manipulation of catalase and superoxide dismutase protein SOD could alter lifespan in the fly [32, 35]. The systemic and cell specific effects of catalase and SOD in the fly are not yet fully clear and adult muscle specific expression of these proteins has not been assayed in homogenized genetic background conditions. Interestingly, recent evidence has demonstrated that genetic manipulation of mitochondrial antioxidants, given by the overexpression of human mitochondrial catalase in old mice, protects from oxidative damage and age-associated mitochondrial dysfunction, together with protecting from energy metabolism diminution in age [36]. In a similar manner, young mice deficient in* Superoxide dismutase 1* (*Sod1*) age prematurely and develop severe sarcopenia [37]. Susceptibility to transient permeability of the outer mitochondrial membrane is higher in rodents without* Sod1* indicating a high apoptotic potential. Furthermore, the levels of the proapoptotic factors Bax and Bak are significantly elevated in mitochondria without* Sod1*, whereas Bcl-XL and Bcl-2 were lowly regulated [8]. These studies highlight how mitochondrial dysfunction can easily be involved in the processes of aging and age-dependent muscle atrophy. To further support this evidence, Umanskaya et al. [36] described improvement of mitochondrial and muscle function by genetically enhancing antioxidant capacity. Using human* mitochondrial catalase* (*mCat*) overexpression, they improved tetanic Ca^2+^ in skeletal muscle, reduced sarcoplasmic reticulum Ca^2+^ leak, and increased sarcoplasmic reticulum Ca^2+^ load in muscle from aged* mCat* mice, thus providing significant evidence for a direct role of mitochondrial ROS in the appearance of pathological signs of sarcopenia. Other hypotheses have emerged as more molecular participants have been described. For example, Diolez et al. [38] proposed the participation of the Adenine Nucleotide Translocator (ANT) in the mitochondria as a new mechanism of protection against increased ROS production in aged skeletal muscle.

Several questions remain open regarding the behavior of ROS during organism and muscle aging. For example, when in lifespan do ROS first appear in the muscle? or which concentrations of ROS are required to alter the gene and protein networks that ensure mitochondria and muscle quality functions? These are still matters to be addressed.

## 3. Mitochondrial Dynamics in Aged Muscle Cells

**Figure 1:**
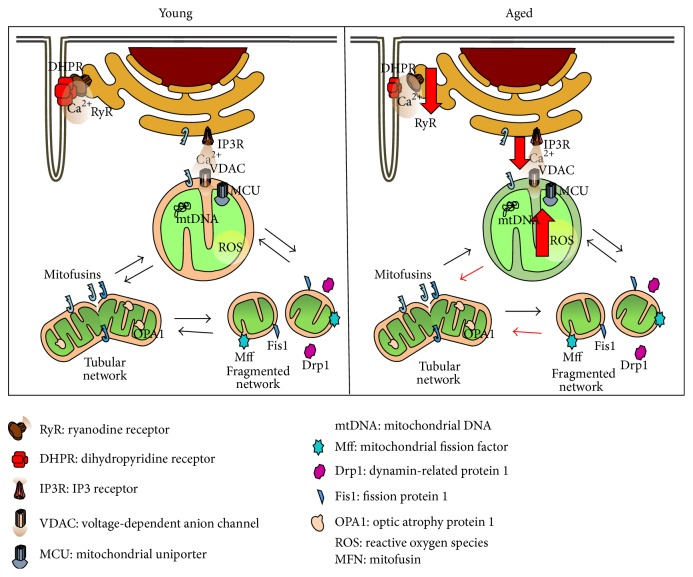
Mitochondrial homeostatic mechanisms altered during aging. (1) Impaired mitochondrial function correlates with excessive ROS production and damaged mitochondrial DNA. (2) Alterations in the excitation-contraction coupling and impaired mitochondrial Ca^2+^ uptake had been found in aged skeletal muscle fibers. (3) Fused phenotype may be increased during aging.

On the past decades, mitochondria, once thought as a single double membrane organelle, have been redefined as a continuous and dynamic interconnected membrane-bound network [48]. Such concept has led to the perception that mitochondrial dynamics plays a more relevant role in cell homeostasis and cell adaptation to the environment than previously acknowledged. Furthermore, this phenomenon appears to be universal throughout the animal kingdom being present in most cells types from yeast to humans. Arrangement and rearrangement of mitochondrial morphology into a dynamic network involve the two key processes of fusion and fission (Figure 1 and Table 1). Fusion is a highly controlled and differentiated process which starts with the outer mitochondrial membrane (OMM) and is followed by the fusion of the inner mitochondrial membrane (IMM). Fusion of the OMM requires a low concentration of GTP, while fusion of the IMM requires hydrolysis of GTP and an intact mitochondrial membrane potential (Cmt) and, therefore, high ATP synthesis [49, 50]. Despite the complexity of the fusion events, evolutionary conserved key players such as the three dynamin-related GTPases, Mitofusin (Mfn) proteins 1 and 2 and the Optic Atrophy-1 (OPA1) protein have been identified [51–53]. In mammals, Mitofusin proteins drive the fusion of the OMM through homotypic and heterotypic interactions of the tubular network, while OPA1 couples fusion of the OMM to the IMM. Loss of either Mfn or OPA1 leads to a mitochondrial fragmentation phenotype. In flies, the first-known mediator of mitochondrial fusion was “*fuzzy onions*” and is expressed specifically in male testes [54]. However, the fly genome encodes a second, ubiquitously expressed mediator of mitochondrial fusion named* dMFN* (*Drosophila Mitofusin*) or* Marf (mitochondrial assembly regulatory factor)* [55]. Ubiquitous downregulation of* Marf* in flies causes second-instar larval lethality, while somatic or cardiac muscle specific downregulation causes a mitochondrial fragmentation phenotype, like in vertebrates [40, 56–58]. An* Opa1* homolog is also present in flies.* Opa1* mutations cause embryonic lethality in flies. Interestingly, like in the case of* Marf* downregulation,* Opa1* specific downregulation in somatic or cardiac muscle leads to a mitochondrial fragmentation phenotype [57, 59]. Furthermore, loss of* Opa1*, assessed using eye specific mutant somatic clone experiments, also induces a mitochondrial fragmentation phenotype, confirming* in vivo* the role of fly OPA1 in the fusion process [57, 60]. On the counterpart, the fission process required to redistribute mitochondria is governed by the activities of the Dynamin-related protein1 (Drp1) and fission protein 1 (Fis1) [61]. Recently, a new player, a C-tail anchored protein named Mitochondrial fission factor (Mff) was identified as an active component of the fission machinery during a* Drosophila* RNA interference library search for mitochondrial morphology alterations in S2 cells [62]. In flies,* Drp1* mutants, mapped by complementation analyses, harbor fewer mitochondria at the neuromuscular junction which affects ATP production at this location leading ultimately to impairment of synaptic vesicle trafficking during prolonged stimulation [41]. Moreover, flies with muscle specific knockdown of* Drp1* using* Drp1* RNA interference or assessing muscle cells of* Drp1* ^*1/2*^ mutants show elongated mitochondrial morphology and increased average mitochondrial area in the tissue [40, 42]. Altogether, studies of mutant model organisms have shed light onto the essential functions of the machinery as well as onto the importance of a proper balance between fusion and fission in order to maintain cell homeostasis and systemic physiology. Nevertheless, further genetic studies using conditional tissue specific mutants and ulterior* in vivo* genetic screens are still missing in order to understand whether there are tissue specific components in the fusion/fission machinery. For instance, underlying mechanisms of the fission/fusion balance in different cell types could be given by tissue specific interactors and regulators of this machinery. Supporting this idea, it has been found that Mfn2 interacts with other Mfn2 molecules or with Drp1 through different regions in the Mfn2 protein to regulate fusion/fission balance [63]. Altogether, these data poses the increasing need to study putative cell specific gene regulatory networks and proteomic networks associated with fusion/fission proteins. In this regard, initial transcriptomic analysis of rats of different ages ranging from 6 to 27 months has shown a significant decrease in expression of mitochondrial fusion/fission related genes* PGC-1α*,* Mfn1/2*,* Fis1*,* Opa1*, and* Drp1*, in 12 months old rats and older age groups, suggesting that mitochondrial dynamics are central in order to maintain muscle quality [39].

The complexity of the mitochondrial network architecture in skeletal muscle fibers has been approached by defining two subgroups of mitochondria depending on their localization, intermyofibrillar and subsarcolemmal, each with different capabilities and functions according to cell ATP requirements [64, 65]. Differences between the two subpopulations are seen especially after periods of muscular inactivity. It is during periods of muscular inactivity when subsarcolemmal mitochondria decrease significantly and production of ROS increases in response to denervation, while intermyofibrillar mitochondria are more susceptible to apoptotic stimuli in this condition [66, 67]. Considering variations in the mitochondrial network during atrophy and aging, Romanello et al. [68] described that the induction of mitochondrial fission by overexpressing fission machinery proteins produces a decrease in the area of skeletal muscle fibers as well as increased autophagy. On the contrary, mitochondrial networks with higher fusion rates dependent on Mfn1 and Mfn2 are found in highly oxidative fibers [69].

The relevance of mitochondrial dynamics to cell homeostasis and to overall organisms' physiology is also illustrated in age-associated diseases [70]. Several cardiomyopathies have been found to increase their risk with age. OPA1 expression is diminished and mitochondria are fragmented in biopsies of patients and rats with heart failure [71]. Further, in flies, interrupting fusion by either OPA1 or MARF suppression provokes cardiomyopathy. Specifically, cardiac knockdown of either protein increases mitochondrial morphometric heterogeneity and induces heart dilation and contractile impairment [57, 72]. In a similar manner, conditional cardiac* Mfn1/Mfn2* double knockout mice die of heart failure due to ventricular dilatation and decreased ejection performance after nine weeks of phenotype induction [56]. In this sense, it is worth noticing that more studies of biopsies of patients with age-associated cardiomyopathies are required. Mitochondrial dysfunction has also been associated with late onset neurological diseases like Parkinson's Disease (PD). Mutations in PINK1 cause a monogenic form of PD. PINK1 is a mitochondrial targeted serine/threonine kinase. Parkin is another protein associated with PD models which has been found through genetic interactions to act downstream of PINK1 and to maintain mitochondrial function in dopaminergic neurons and thoracic flight muscles in the fly [73]. Genetic manipulation experiments in* Drosophila* mutant models for* Pink1* or* parkin* show that the thoracic muscle degeneration and locomotor deficit can be rescued by promoting fission of mitochondria via expression of* Drp1* or ablation of* Opa1* in these cells [74, 75]. These studies indicate the crucial role of the mitochondrial proteins PINK1 and Parkin in maintaining proper mitochondrial dynamics to preserve muscle integrity in an age-related disease. Pharmaceutical targeting of muscle PINK1 may result in good palliatives for the movement related symptoms observed in PD patients. Alterations in mitochondrial morphology have also been described in skeletal muscle during age-associated metabolic diseases such as diabetes and obesity. In obese patients, Mfn-2 expression is reduced [76]. Consistently, in skeletal muscle fibers of mice fed a high fat diet, Mfn-1 and Mfn-2 but not Opa1 were decreased, and the proteins governing mitochondrial fission Fis1 and Drp1 were increased [77, 78]. In this sense, mitochondrial dynamics in muscles appears to be sensitive to changes in cell metabolism. Perturbation of mitochondrial dynamics during aging and in age-associated diseases appears to affect muscle quality contributing to the deleterious symptoms in these phenotypes. Such perturbations appear to depend on the expression levels of the conserved key players* Mfn1/2*,* Fis1*,* Opa1*,* Drp1*, and* PGC-1α*. Nonetheless, little is known about the upstream stimuli and regulators which trigger mitochondrial dynamics. Observations from flies and mice suggest that it is the balance of fusion and fission rather than one event or the other that it is important to maintain a healthy aging. Further, from the data available at the moment from model organisms, it is tempting to speculate that movement or exercise and diet could be upstream regulators governing the mitochondria dynamics balance and even constitute a positive feedback mechanism for healthy aging.

## 4. Mitochondrial Calcium Regulation in Aging Striated Muscle

Muscle fibers are well-organized and compact cells, the Endoplasmic Reticulum (ER) and mitochondria networks are strategically localized to supply energy and quality control in muscle cells. Moreover, both ER and mitochondria undergo constant remodeling in response to cellular demands [78], changing their architecture but maintaining their organized disposition in the skeletal muscle fibers. In the recent years, the description of Mitochondrial Associated Membranes (MAMs) has been the target of new research. ER and mitochondria associated proteins have been identified and form the basic components of the MAMs. These proteins include Ca^2+^ ion channels located at the ER or at the outer mitochondrial membranes (OMM) like the Inositol 1,4,5 trisphosphate Receptor (IP3R) [79] and voltage-dependent anion channel 1 (VDAC1) [80], enzymes of the lipid biosynthetic pathways, and lipid transfer proteins [81], as well as various chaperones like the Glucose-regulated protein 75 (Grp75) [82]. Noteworthy is the fact that most of these proteins are evolutionary conserved in flies and mice suggesting similar operating mechanisms. Sequence comparison and biochemical assays have demonstrated that* dry* is the sole ryanodine receptor of the fly [83],* dip* codes for the only* Drosophila* IP3R [83], and PORIN has analogous functions to vertebrate VDAC1 [84]. VDAC1/PORIN fly mutants show locomotive defects and elongated mitochondria. This phenotype can be enhanced by increased mitochondrial fusion or ameliorated by overexpression of fission protein Drp1, demonstrating that directly or indirectly VDAC/PORIN function affects mitochondrial remodeling in flight muscles. Likewise, the fly chaperone Hsc70-5/Mortalin, related to the vertebrate Grp75, has been identified as a regulator of mitochondrial morphology and cellular homeostasis in a proteomic screen for OPA1 interactors [85]. Hsc70-5/Mortalin is an enhancer of OPA1 since its downregulation leads to phenotypes of fragmented mitochondria with reduced membrane potential.

Among other functions, MAMs have been described to be important Ca^2+^ bridges between the ER and the mitochondria and thus may be directly involved in regulating mitochondria oxidative metabolism, cell death pathways [86], and muscle excitation-contraction coupling [87] (Figure 1). Important Ca^2+^ regulator proteins settled in the MAMs like IP3R, and VDAC and the uniporter mitochondrial Ca^2+^ channel (MCU) are well described as the interchanger axis between ER and mitochondria. The entry of Ca^2+^ into the mitochondria occurs through MCU, which is strategically located in the inner mitochondrial membrane [88]. In addition to this unique location, the MCU has low affinity for Ca^2+^, thereby preventing its entry into the mitochondria under normal cytoplasmic concentrations. It is at this point that the location of the organelle becomes important. Once Ca^2+^ enters into the mitochondria, it is used by various enzyme systems, namely, Pyruvate DeHydrogenase (PDH), Glycerol-3-Phosphate DeHydrogenase (G3PDH), isocitrate dehydrogenase, and oxoglutarate dehydrogenase, as a cofactor for Krebs cycle reactions, thus contributing to the maintenance of mitochondrial metabolism [89]. Moreover, blocking the entry of Ca^2+^ into mitochondria significantly decreases cell metabolism [90], whereas prolonged and excessively elevated mitochondrial Ca^2+^ impairs mitochondrial function due to dissipation of the mitochondrial membrane potential and increased ROS production [91]. In short, a suitable mitochondrial function is related to efficient communication with the ER and Ca^2+^ transfer between the two organelles. While a role for mitochondrial dysfunction and decreased metabolism in aging muscle has been extensively described, evidence about the participation of mitochondrial calcium uptake from the ER and the IP3R-VDAC-MCU axis is still lacking. Recent data from Mammucari et al. [92] positively showed that, in mice, mitochondrial Ca^2+^ handling by the MCU regulates skeletal muscle mass through signaling pathways involving protein kinase B (Akt) and PGC-1*α*4. Moreover, overexpression of MCU in skeletal muscle fibers protects from denervation-induced atrophy [92]. Altogether these data suggest that mitochondrial Ca^2+^ regulation is highly involved in anabolic pathways and could effectively control muscle loss. Furthermore, defective sarcoplasmic reticulum- (SR-) mitochondria crosstalk has been causally linked to the abnormal mitochondrial Ca^2+^ uptake in failing hearts and may underlie their increased oxidative stress [93]. Fernandez-Sanz et al. [94] described that, in digitonin permeabilized cardiomyocytes from young hearts, induction of SR Ca^2+^ release with caffeine was followed by a rapid increase in mitochondrial Ca^2+^ uptake and this increase was severely decreased in cardiomyocytes from old hearts. Likewise, in fruit flies, downregulation of MARF in the heart leads to a phenotype with increased contraction-associated and caffeine-sensitive Ca^2+^ release, showing that there is a physical association of SR and mitochondria which occurs through MARF/Mfn2 and is essential for normal Ca^2+^ signaling in cardiomyocytes [47] (Table 1).

In skeletal muscle, the tight link between Ca^2+^ and EC-coupling has also been the focus of research (Figure 1). Impaired EC-coupling function in aged muscle results in a reduced supply of Ca^2+^ ions to the contractile elements and, thus, reduced specific force [43]. These findings correlate with previous studies describing impaired Ca^2+^ release in aged muscle due to a decrease in Cav1.1 (dihydropyridine receptors, DHPR) [44] and reduced SR Ca^2+^ release in SR vesicles [45, 46], possibly by uncoupling between the voltage sensor (DHPR) and ryanodine receptors (RyR1) which may also contribute to muscle weakness in aging. Pietrangelo et al. [95] proposed age-dependent uncoupling of mitochondria from the Ca^2+^ release units. They described an increase in damaged mitochondria together with reorganization of the SR-mitochondria interaction, with a significant decrease in tethers and misplaced mitochondria from the normal triad position, possibly resulting in reduced metabolic efficiency and a consequent decline of skeletal muscle performance. Enhancement of antioxidant activity of mitochondria results in an improvement of aged mice muscle function due to decreased intracellular Ca^2+^ leak and increased SR Ca^2+^ load. In these same experiments, there were differences in the RyR oxidation levels between young and old mice when submitted to genetically enhanced antioxidant activity [36]. Compatible with these observations, recent data suggests that SR Ca^2+^ leakage and accumulation occurs in muscle type I fibers of old subjects as a consequence of reversible oxidative modifications of RyRs [96]. On the basis of these findings, it is valid to speculate that disturbances in Ca^2+^ homeostasis towards the EC-coupling with the consequent perturbation of mitochondrial metabolic function precede the appearance of sarcopenia.

## 5. Mitochondrial Biogenesis and Mitophagy

Mitochondrial dynamics not only is governed by fission and fusion processes but also implies processes of mitochondrial biogenesis and degradation. The balance between these last two processes gives the cell new pools of mitochondria and confers mitochondrial quality control. Deficiency in mitochondrial fission processes during aging could promote mitochondrial dysfunction and a subsequent accumulation of damaged mitochondria. In fact, confocal microscopy experiments following mitochondria into autophagolysosomes and mitochondrial potential registration point to mitochondrial fission processes preceding mitophagy to maintain homeostasis of these organelles [97]. To unveil the role of biogenesis in the aging process, Joseph et al. [98] used a mouse model of premature aging (PolGmutator) and compared it with normal aged mice. They observed that mtDNA mutations could alter mitochondrial morphology producing an increase in fusion and contributing to sarcopenia. Moreover, these mice differed in the expression of mitochondrial proteins. Mfn1 and Mfn2 levels were significantly higher with normal aging, while Fis1 levels were reduced in older animals when compared to young animals, indicating higher levels of fusion in muscle of physiologically aged mice. By contrast, Fis1 levels were higher in muscle from older PolG animals when compared to age-matched wild-type mice. The divergent response in muscle highlights the diversity and complexity of the underlying mechanisms involved in skeletal muscle pathologies compared to aging.

In* Drosophila*,* Spargel* (*srl*) the homolog of PGC-1*α* coordinates mitochondrial biogenesis in fat bodies in response to insulin [99]; however, little is known about* srl* role in the aging muscle. More about the role of PGC-1*α* is known from mouse skeletal muscle. Several studies have reported that an increase in PGC-1*α*, the major regulator of mitochondrial biogenesis, may suppress atrophy symptoms [100, 101]. Overexpression of PGC-1*α* in skeletal muscle induces a fast (type II) to slow (type I) fiber type conversion and increases mitochondrial content and oxidative capacity through the modulation of genes involved in metabolism like* Citocrome c*,* COXII,* and* COXIV* [102]. Oppositely, sarcopenia apparently begins with a diminution of type II fibers and a fissioned mitochondrial network with punctual mitochondria and short mitochondrial domains in these type of fibers [103]. On the other hand, Cannavino et al. [100] reported that the overexpression of PGC-1*α* effectively prevented muscle disuse-induced atrophy. Further studies have unveiled a strong relationship between PGC-1*α* and mitophagy, suggesting a dual role for this protein in mitochondrial turnover [101, 104]. Animals lacking PGC-1*α* exhibited less mitochondrial population together with autophagy deficient mechanisms and altered muscle phenotype [101]. Induction of autophagy and lysosomal protein expression, mediated by denervation in wild-type animals, was partly attenuated in PGC-1*α* KO animals, resulting in reduced autophagy and mitophagy flux.

Considering that aging develops an inflammatory scenario, one line of evidence suggests that inflammation could directly affect mitochondrial clearance and further enhance aging effects on skeletal muscle through the macrophage migration inhibitory factor and the subsequent inhibition of mitochondria dependent death pathways [105].

## 6. Perspectives

To date, only a few genetic interactors have been found to drive mitochondrial function and dynamics. With the refinement of transcriptomic and proteomic techniques, it has become a pressing matter to elucidate the gene and protein networks controlling function and dynamics of mitochondria in order to find new players of these organelles homeostasis. The role of model organisms, in particular* Drosophila*, will be crucial for* in vivo* validations of these high throughput networks and for the study of the evolution of these networks throughout lifespan. Further insights into the mechanisms that govern the aging process and how muscle quality contributes to the overall organism homeostasis will come from the understanding of whether these regulatory networks are tissue specific and when in lifespan are they perturbed.

Currently, exercise is the only known effective method to stop muscle degeneration. Thus, future research should address the question on how genes and proteins from the mitochondrial network are expressed during exercise and how do these changes correlate with the functional capacity of skeletal muscle fibers. Further, in vertebrates, it is becoming apparent that mitochondrial dynamics are tailored to the fiber type metabolic status, indicating that there might be differential regulators of mitochondrial homeostasis in different fibers. Perturbations in these gene/protein interaction networks could lead to the finding of key fiber specific regulators which may be used as therapeutic targets for sarcopenia and muscle degeneration.

## Figures and Tables

**Table 1:** Summary of the evidence for mitochondrial homeostatic mechanisms altered during aging of flies compared to mice.

		Mice		Flies
Oxidative stress	↑	ROS [15] Affects the complex V (ATP synthase) of the ETC [21]	↑	ROS [22, 35]

Antioxidant systems	↓	Diminished ROS scavengers' activity during aging [33] Overexpression of human mitochondrial catalase in old mice protects from oxidative damage and age-associated mitochondrial dysfunction [36]	↓	Diminished ROS scavengers' activity during aging [32, 34] Genetic manipulation of catalase and superoxide dismutase protein, SOD, could alter lifespan in the fly [32, 34]

mtDNA		Increase of 8-oxodeoxiguanosine (8-oxoG), indicating mtDNA oxidation Alteration of mtDNA copy number in muscle cells [24, 29, 30]. mtDNA haplotype mutation arise in early life [26]		Naturally occurring variations in mtDNA influence mitochondrial bioenergetics [22, 23] Alteration of mtDNA copy number in muscle cells [22, 23] mtDNA haplotypes may correlate with lifespan [23]

Mitochondrial dynamics		Enlarged mitochondria in aging muscles Mitochondrial fusion/fission genes show altered expression in old animals [39]		*Drp1* mutants harbor fewer mitochondria at the neuromuscular junction [40] *Drp1* muscle knockdown shows alterations in mitochondrial morphology and distribution [41, 42]

Ca^2+^ regulation		Impaired EC-coupling [43]. Reduced supply of Ca^2+^ ions to the contractile elements Age-dependent uncoupling of mitochondria from the Ca^2+^ release units [43–46]		Heart tubes deficient of MARF (dMFN) had increased contraction-associated and caffeine-sensitive Ca^2+^ release, suggesting a role for MARF in SR Ca^2+^ handling [47].

ETC: Electron Transport Chain; ROS: Reactive Oxygen Species; EC: Excitation-Contraction coupling; mtDNA: mitocondrial DNA; Drp1: Dynamin-related protein 1; MARF: Mitochondrial Assembly Regulatory Factor; dMFN: Drosophila Mitofusin; SR: Sarcoplasmic Reticulum.
